# Idiopathic pulmonary fibrosis diagnosed concomitantly with diffuse squamous cell lung cancer on surgical lung biopsy: a case report

**DOI:** 10.1186/s13256-021-03177-7

**Published:** 2021-12-15

**Authors:** C. Niel, C. Ricordel, T. Guy, M. Kerjouan, B. De Latour, D. Chiforeanu, M. Lederlin, S. Jouneau

**Affiliations:** 1 Department of Respiratory Medicine, CHU Rennes, Rennes 1 University, Rennes, France; 2 INSERM U1242, Chemistry Oncogenesis Stress and signaling, CLCC Eugène Marquis, Rennes, France; 3 Department of Respiratory Medicine, Centre Hospitalier Bretagne Atlantique, Vannes, France; 4 Department of Thoracic Surgery, CHU Rennes, Rennes 1 University, Rennes, France; 5 Department of Anatomopathology, CHU Rennes, Rennes 1 University, Rennes, France; 6 Department of Radiology, CHU Rennes, LTI, INSERM U1099, Rennes 1 University, Rennes, France; 7 IRSET UMR 1085, Rennes 1 University, Rennes, France

**Keywords:** Idiopathic pulmonary fibrosis, Squamous cell lung cancer, Surgical lung biopsy, Nintedanib

## Abstract

**Background:**

Idiopathic pulmonary fibrosis is a disease with a poor prognosis and has been associated with increased lung cancer incidence.

**Case presentation:**

We report the case of a Caucasian 75-year-old woman, a former smoker, hospitalized for breathlessness with a chest computed tomography scan showing an interstitial lung disease. A surgical lung biopsy was performed, confirming a pattern of usual interstitial pneumonia but also numerous disseminated foci of well-differentiated focally invasive squamous cell carcinoma without hypermetabolic lung nodule, mass, or enlarged lymph node visualized on chest computed tomography or positron emission tomography scan. Nintedanib was started for its antifibrotic and antitumor properties, without any other antineoplastic treatment. Three years after initiation of nintedanib, clinical, functional, and computed tomography scan evaluations were stable, and there was no evidence for evolution of the squamous cell carcinoma.

**Conclusions:**

Data are scarce regarding the benefit of nintedanib in patients with idiopathic pulmonary fibrosis-associated lung cancer, and it is unclear whether nintedanib could have a preventive role in lung carcinogenesis in idiopathic pulmonary fibrosis patients. This experience could help the scientific community in case of similar incidental findings.

## Background

Idiopathic pulmonary fibrosis (IPF) is a poor-prognosis disease of unknown origin. According to epidemiological studies, up to 22% of patients with IPF will develop lung cancer, with a nearly five times increased risk compared with the general population [1]. Even if the link between lung cancer and IPF has been extensively studied, no clear common pathogenesis mechanism has been confirmed to date [2]. We report herein the case of a patient with IPF diagnosed by surgical lung biopsy, which concomitantly revealed a diffuse squamous cell lung cancer (SCC). Follow-up at 3 years after nintedanib treatment showed no progression of the two diseases.

## Case presentation

**Fig. 1:**
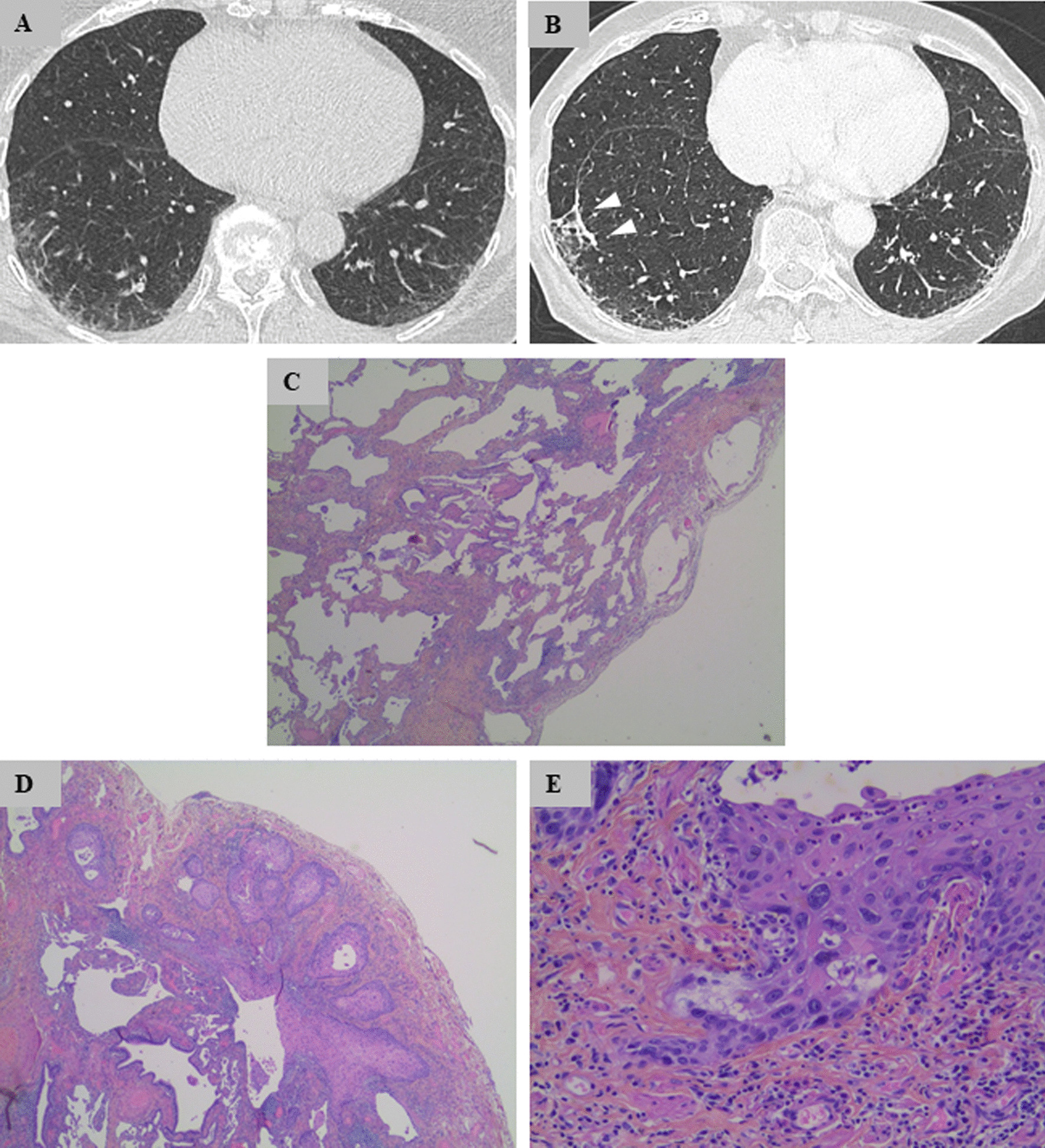
**A**, **B** Chest computed tomography in lung windows showing posterior subpleural reticulations at baseline **A** with no significant change at 2-year follow-up (**B**). Arrowheads represent the line of biopsy staples. **C** Idiopathic pulmonary fibrosis lung tissue showing fibroblastic foci and honeycombing [hematoxylin–eosin–saffron (HES) ×50]. **D** Well-differentiated SCC (HES ×200). **E** Microinfiltrating SCC (HES ×400)

In November 2016, a Caucasian 75-year-old woman, a former smoker (7.5 pack-years), was hospitalized for breathlessness. Her past medical history included atrial fibrillation treated with warfarin, arterial hypertension treated with betaloxol, and gastroesophageal reflux. She ran a bar–tobacco shop with significant long-term exposure to passive smoking. She was not exposed to asbestos. Clinical examination on admission revealed good performance status, stage 1 modified Medical Research Council (mMRC) dyspnea, crackles at lung bases, and no digital clubbing or extrathoracic signs. Chest computed tomography (CT) scan showed interstitial lung disease (ILD) with subpleural reticulations without evidence of honeycombing or enlarged lymph node (Fig. 1). Standard biology, serological testing, bronchoscopy, and bronchoalveolar lavage were normal. Pulmonary function tests demonstrated bronchial obstruction [forced expiratory volume in 1 second (FEV1)/forced vital capacity (FVC) ratio, 0.66; FEV1, 97% of predicted values (% pred.)], preserved volumes [FVC, 121% pred.; total lung capacity (TLC), 111% pred.], and alteration of gas diffusion (TLCO, 62% pred.). ILD multidisciplinary discussion (MDD) reached a CT pattern of possible usual interstitial pneumonia (UIP), which led to the proposal of performing surgical lung biopsy. Wedge resection of the right upper and lower lobes was performed by video-assisted thoracoscopic surgery in July 2017. Both resected lung specimens showed a similar pattern of UIP: fibroblastic foci and honeycombing. In addition, the right lower lobe specimen displayed numerous disseminated foci of well-differentiated focally invasive SCC without invasion of visceral pleura (Fig. 1). Complete resection was obtained without SCC-positive surgical margins. A PET–CT scan performed in September 2017 showed mild hypermetabolism of ILD [maximum standardized uptake value (SUV_max_), 3.5], without hypermetabolic lung nodule, mass, or enlarged lymph node. Cerebral magnetic resonance imaging (MRI) was normal. There was no indication for adjuvant antineoplastic treatment based on pathological findings. After ILD MDD in October 2017, it was decided to introduce an antifibrotic treatment owing to pathological confirmation of UIP. Nintedanib was chosen as this molecule is also known for its antitumor properties. At the last follow-up in October 2019, that is, 2 years after initiation of nintedanib, clinical, functional (FVC, 130% pred.; TLCO, 61% pred.), and CT scan evaluations were stable (Fig. 1). There was no evidence of squamous cell carcinoma progression.

## Discussion

Idiopathic pulmonary fibrosis (IPF) is a chronic, progressive disease associated with significant morbidity and mortality. IPF lesions seem to represent an independent risk factor for lung carcinogenesis [3]. IPF has been shown to increase the risk of lung cancer, ranging from 3% to 22% [4]. Both diseases present similar pathogenetic features, notably the involvement of multiple growth factors, and a possible genetic predisposition for lung cancer in IPF patients.[5] In our case, squamous metaplasia and fibroblast foci were observed within the fibrotic lung pathological specimen. Some authors suggest that it may represent an early feature of tumorigenesis [2]. Interestingly, the diffuse SCC was diagnosed in the lower right lobe in our case, in accordance with what is known from cohort studies [6]. This observation emphasizes the connection between IPF-induced structural damages and SCC occurrence. IPF patients diagnosed with lung cancer have a poorer prognosis than patients with IPF alone. Moreover, lung cancer treatment is associated with significant toxicity, especially in the context of IPF. Surgical intervention, chemotherapy, and radiation therapy can induce an exacerbation of IPF and be detrimental for patients [3]. Considering the similarities of pathogenesis pathways between these diseases, some antiproliferative agents, such as nintedanib, have been tested [5]. Nintedanib is an intracellular multikinase inhibitor targeting growth factor receptors [such as fibroblast growth factor receptor (FGFR), vascular endothelial growth factor receptor (VEGFR), platelet-derived growth factor receptor (PDGFR)] [7]. Efficacy and safety of nintedanib in patients with IPF was demonstrated in INPULSIS trials. These randomized, placebo-controlled, phase 3 trials showed that nintedanib significantly reduced the annual rate of decline in FVC [8], and are now recommended for the treatment of mild to moderate IPF [9]. Nintedanib is also an anticancer agent and has proven its efficacy in LUME trials. In both studies, the combination of nintedanib with docetaxel or pemetrexed improved progression-free survival in second-line treatment of non-squamous non-small cell lung carcinoma (NSCLC).[10, 11] There are no data on the benefit of nintedanib in patients with IPF-associated lung cancer (J-SONIC trial is still ongoing [12]) or on possible prevention of lung cancer in IPF patients.

## Conclusion

We report a case of IPF diagnosed on surgical lung biopsy, associated with diffuse SCC without any nodule, mass, or lymph node found on chest CT or PET–CT. This observation emphasizes the connection between IPF-induced structural damage and SCC occurrence, both found in the subpleural lung region. After 3 years of nintedanib treatment, which was administered for its antifibrotic and antitumor properties, both IPF and SCC show no sign of progression. Scientific data are still scarce regarding the benefits of nintedanib in patients with IPF-associated lung cancer, and it is unclear whether nintedanib could have a preventive role in lung carcinogenesis in IPF patients.

## Data Availability

All data generated or analyzed during this study are included in this published article.

## Abbreviations

IPF: Idiopathic pulmonary fibrosis
SCC: Squamous cell lung cancer
ILD: Interstitial lung disease
MDD: Multidisciplinary discussion
UIP: Usual interstitial pneumonia
NSCLC: Non-squamous non-small cell lung carcinoma
